# Address

**Published:** 1872-07

**Authors:** 


### ADDRESS.

*Gentlemen of the Graduating Class:*

It is by the appointment of the Medical Faculty of this University that I have the honor of addressing you upon this interesting occasion. Always proud of any opportunity of acting as their representative, I esteem myself peculiarly fortunate at this time, when the object is to welcome your entrance into the ranks of our profession. The pleasure ordinarily afforded is greatly lessened, however, by the painful conviction of inability adequately to discharge the important trust thus imposed; and in an humble effort to fulfill the task assigned me by the partiality of my colleagues I confidently claim from them and yourselves the same indulgent forbearance which has been extended to all my labors.

Permit me then, gentlemen, in behalf of my colleagues, and for myself, to express a cordial sympathy with you in this most important era of your lives, and to detain you with a few desultory remarks relative to your future career, before our final separation. We have brought our arduous and important labors to a close, and are about to take leave of each other. Our familiar greetings and interviews, the efforts to impart and receive instruction, must now terminate. To me these separations, these annual partings—landmarks in the pathway of life—beacons on the passage to the grave—are full of interest, in which pleasure is commingled with sadness. Saddened I confess I am at the reflection, that this is our last interview in our present relations, relations in which I have so many reasons for the expression of grateful emotions, for the considerate respect and forbearance extended to my imperfect efforts. I have felt conscious that your sympathies were with me, and it has cheered me in my undertakings, and inspired me with delight and confidence, and their interruption will produce unfeigned regret.

On the other hand, it affords us sincere pleasure to know that you have grown in knowledge beyond the need of our assistance, and that you are about to take your flight into the world of action, each trusting to his own wings, and selecting his own course in the broad expanse before him. In our sympathy with you at this time, we live over again one of the happiest and most exciting moments of our own existence. The whole picture of your emotions is indelibly traced upon our memory. We participate in the proud satisfaction of your retrospective view, in the delight of your present relaxation from toil and anxiety, in the buoyant gladness of your new independence, in the lofty aspiration, the hope, the confidence, the joy of your eager glance into the future. The congratulations of your preceptors, therefore, on this occasion are not the mere expressions of cold formality; they are the heartfelt overflowings of a real participation in your feelings, and of a true interest in your welfare.

One parting word of counsel, dictated by such motives will, I am sure, be received in the same spirit of kindness in which it is given.

In the very outset, then let me exhort you to imprint indelibly upon your minds the conviction that your success in life will depend mainly upon yourselves. Trust nothing to fortune or to the fancied advantages of your position. Labor diligently, in your intervals of leisure, to render yourselves more competent for the performance of your professional duties; guard your sentiments and conduct so as to command the respect of honorable men; and endeavor to cultivate such an exterior deportment as may render your presence acceptable to those whose society you may desire. Thus equipped, if you watch diligently the current of affairs, neither imprudently rushing into the midst of adverse events, nor allowing any favorable opportunity for honorable action to pass unimproved, you will as certainly prosper in your professional undertakings, as the seed sown in a good soil, and nurtured with due care, will spring up and ripen into harvest. The moral world is governed by laws scarcely less uniform in their operations than those which regulate the physical. Much less is justly ascribable to accident than young men are usually disposed to imagine. The successful often feel a pleasure in considering themselves the favorites of fortune, while the unsuccessful are ever willing to shift off from their own folly or carelessness the responsibility of their failure; but there are few men so purely fortunate as to be unable to point to some prudent forethought, or wise decision, as the real origin of their success, while perhaps not one thriftless, disappointed man can be found who cannot recall numerous instances in his experience, of time misspent annd opportunities neglected. Keep this maxim always before you, that under Providence you must rely chiefly upon your own unaided efforts, and with the inflexible purpose of leaving no honorable means untried of promoting your advancement, you cannot fail of obtaining, if not the summit of your ambition, at least a respectable station in life, with a competent provision against all ordinary calamities.

Not that I desire your attention to be exclusively occupied with your own worldly prosperity. It is not necessary that the whole channel of your soul be compressed within the turbid and narrow limits of selfishness. By a wise ordinance of Providence the exercise of an expanded benevolence is not incompatible with our true interests. It turns away the thoughts for a moment from the schemes of profit or ambition, and more than repays the loss by its cheering effect upon the heart, and its ennobling influence upon the character. "The overflow of kindly feeling, at the same time that it enriches the soil upon which it spreads, purifies and sweetens the stream from which it proceeds, and to which it again returns." If actuated, therefore, by no higher motive than a regard for our own happiness, we should cultivate good will to others, multiply friendly relations with objects around us, and throw out in all directions the cords of endearing associations, by which we may reciprocally draw and impart refreshing sympathy and useful support. In the future prosecution of your professional labors you will find that medicine, when rightly pursued as a calling, is by its very nature beneficent; its constitution is charity, its aim to rescue the afflicted, to suspended or avert the stroke of death, to restore to health and strength, and ease the victim of sickness, debility or pain.

The first application which I shall desire you to make of the habits of industry which you have been recommended to cultivate, is in the continued prosecution of your professional studies. It is true that this day is the closing scene, the crowning result of your course of collegiate study. But I need scarcely say to you that you can add to the stock of knowledge which you now possess of the healthy and morbid movements of that wonderfully complicated machine, the nature of which has been for a few years past the object of your earnest inquiry. It has indeed been well remarked that the moment a practitioner ceases to be a student, he is no longer worthy the confidence of the public. The life of a physician can only be truly useful and honorable when it is unremittingly employed in determining the truth of theoretical opinion by observation; and in improving the value of practical suggestions by the test of experience. You must not be idlers; stand still and you are left far behind; swim on the flood of improvement or you will be cast on the shore among the useless rubbish. No matter what may be your abilities or attainments, they cannot be developed without a certain degree of reflection and mental discipline, and although this amount may vary according to the precise capability, no marked development can occur in any case without study. The extensive improvements which are constantly taking place in the different departments of medical science render it utterly impossible for any practitioner to pursue his avocation with satisfaction to himself unless he bestows upon every case that demands it a degree of scrutinizing investigation, which is needless to the empyrical pretender. Ignorant of the operations of the animal economy, he prescribes recklessly, and should the morbid condition become aggravated, he sees not the connection between the agent employed and the mischief effected. Remaining in a state of blissful ignorance, he consoles himself perhaps that his patient has fallen a victim to a malady, the fatal effects of which no human efforts could have averted. How blissful indeed compared with the anxiety that rests upon the mind of the philosophical practitioner. Aware of the manner in which the functions of the economy are executed in health; equally aware of the aberrations that constitute the diseased manifestations; instructed from his own observation, as well as from the recorded observations of others, of the results which his various therapeutic agents are capable of effecting, he weighs carefully and hesitatingly the symptoms that guide him to the formation of his indications of treatment. Sensitively alive to the safety and welfare of his patient, and to the relief of his sufferings, what anxiety does he not endure in the application of his remedial measures? What mental uneasiness does he not experience until he finds that the threatened danger has passed away?

Much is said in modern times in our profession about practical knowledge and practical men, and an effort is sometimes made to establish the false position that he who reads attentively and studies closely cannot be a practical man. There is, unquestionably, a certain tact, a certain faculty of invention and adaptation, a certain amount of common sense which some men do not possess, and without which one cannot become a great practitioner, though he may be well versed in science; but, be assured, the error here lies in the want of this tact, and not in being over-learned. If he knew less, he would be still less capable of practice. Be not deceived by the fact that there may be some indolent men of such native vigor of mind, tempered so justly with common sense, and sharpened by shrewd observation, that without the ordinary literary and scientific attainment, they are enabled to avoid great mistakes, or possibly attain a degree of respectability in the profession. May we not rather ask, how much more well-directed, industrious application of a mind thus gifted to the different sciences pertaining to medicine would have accomplished?

Look into the lives of all the great masters of our art, all who illuminate the history of the past, and you will find them acquainted with the science of their day, and the principles of the art; not mere practical men, if that term be used as synonymous with ignorance. Indeed, gentlemen, I have never known theoretic attainment lightly estimated except by those who did not possess it. Influenced by such opinions it has afforded me unfeigned pleasure in my own department, to witness on your part, a determination to learn the science of medicine before you should attempt to practice the art. True, experience is necessary; clinical observation is, I admit, necessary to the attainment of professional eminence. And, gentlemen, you will profit by your bedside observation precisely in proportion as you have become equipped with scientific armor, which alone will enable you to comprehend what you see. Does not the nurse see disease all her life with no other result but to be self-sufficient? and yet you often meet with practitioners of medicine who boast of a long and extesive experience, having never learned anything, for the simple reason that they did not possess enough science to furnish them a key to the comprehension of what was passing under their eyes. Here, then, was the place for you to learn the science of medicine — the practice you will be learning all the rest of your lives — and you will profit by the latter precisely in proportion as you are well grounded in the former.

Enlightened self-interest constrain us to this course. By so doing only can the practice of medicine be made attractive. He who truly loves his profession and its study, with these just views relative to the manner in which it should be undertaken, possessing the tact and quick discernment which it requires, added to a thorough education, will experience a constantly increasing happiness in studying to improve and exercise thereby his intelligence and reason. The man who pursues the practice of medicine as a mere trade or means of acquiring a living is greatly to be pitied, as he is doomed to drag out a miserable existence, and with the certain result of professional insignificance, and probably of pecuniary disappointment.

By pursuing this enlightened course of constant study only can the practice of medicine be made attractive. When indolence is thrust aside and mercenary motives denied entrance, and we look at medicine simply as a science, it is full of interest, and the study of it is therefore a rich source of gratification. Its subjects are comprehensive in extent and endless in variety. It is intimately connected with all the other sciences. It gathers to itself the resources of chemistry, botony, minerology, comparative anatomy, and physiology; mental philosophy is an important auxiliary, and it levys a heavy contribution upon mechanics and thus fills its storehouse with a variety and abundance sufficient to satisfy the largest and most eager curiosity.

The phenomena of life, even in the healthy condition, are exceedingly diversified, as modified by disease, and by the remedies, which may be administered, their variations are never ending. Superadd the mysterious connection of mind and body, and not only is the field of contemplation still more varied, but a mass of facts of a mingled mental and physical character is opened to the observer, which awaken an intense interest. We often perceive an absorbing enthusiasm manifested in the pursuit of medical science not common in other studies, an enthusiasm which leads its votaries to disregard the loathsomeness of putrefaction, to leave unheeded all the dangers of contagion and infection, and in search of truth, becoming, in some instances, utterly unmindful of their own comforts or necessities.

In addition to a careful perusal of standard works, keep yourselves *au courant* with professional progress by reading the medical publications and periodical journals of the day. Not that I would have you adopt, untried, the various novelties or theories of the hour, but there should be nothing so new that you are not familiar with what is known on the subject. In this way only will you avoid routinism—one of the dangers to which the isolated practitioner is especially exposed.

Whilst your professional studies and engagements should first claim your attention, your general reading should by no means be neglected. You do not return to your several places of abode again to become private citizens. You must now take your position in society as public characters; the public eye will be upon you; your intelligence will be measured by a high standard. You will be expected to act creditably your parts to furnish your quota of instruction at home and abroad, to be useful citizens and agreeable neighbors.

The community will no longer believe a man a genius, possessing an innate knowledge of setting bones or compounding medicines, but will require, before they bestow upon him their confidence, that he possess a respectable share of general intelligence and good judgment. Besides, the exclusive exercise of a single faculty blunts the remainder. Do not, therefore, confine your reading to that which is strictly professional. All studies, properly pursued, are capable of yielding pleasure and advantage; still, as every one must be content to be ignorant of much, it is important that each make a selection of what befits his studies and his profession as well as his partialities. Again, whatever is worth reading at all, is worth reading understandingly, else a habit of superficial reading is acquired, which unfits the mind for reflection and appreciation. The mind becomes obtuse and we read to no purpose, when once this habit is established.—A blaze of light will not enable the blind to see, nor perspicuity make the thoughtless understand. Occupy your leisure hours, then, with your books and keep yourselves prepared to discharge creditably the duties which may devolve on you in any emergency. Exercise, a rigid discrimination, however, in the selection of your unprofessional reading. Possibly, straitened means or want of time have prevented your acquiring, whilst pursuing your medical studies, that knowledge of history, of biography, of polite literature, and, above all, that acquaintance with the various events which have transpired in the Republic in which you have the duties of citizen and sovereign to perform, all of which are necessary accomplishments to a well-bred gentlemen; and, if so, avail yourself of the leisure which you will most likely have at command during the first few years of your practice to supply this deficiency. Do not understand me as deeming these remarks necessary from any imperfections which have been discovered as specially pertaining to those included among the members of the present class. No. We all require daily to renew our good resolutions, and to be stimulated to increased efforts by the good example and encouraging words of those around us, and in this spirit only it is desired these remarks will be accepted. Indeed, I should not deal honestly with myself, did I not admit that I have had the pleasure of seeing assembled in the halls of the University of Buffalo, during the past season, the most intelligent body of young men with which it has ever been my fortune to be associated. With such illustrations and proofs of the adequacy of the means of instruction afforded by this institution no fears need be entertained of the ultimate realization of the most complete success which its most ardent friends could desire.

With honest pride may the faculty of their appointment ask those generous citizens who have so liberally contributed to the permanent establishment of this, the first enterprise of the kind ever undertaken in this great commercial city, here to behold the fruit of the tree which they have planted, as conclusive evidence that their beneficence has been judiciously bestowed. Would that the prosperous condition of the medical department, whose ultimate triumph has long since been placed beyond a contingency, might arouse a spirit of liberality in the citizens of Buffalo which alone is necessary to accomplish that great desideratum—the organization and establishing on a permanent basis the academic department of this university.

From you, young gentlemen, it may be added, shall we be content to receive the measure of our success, having full confidence in your integrity and capacity, and believing that you will at least have perceived an honest desire to spread before you plain practical truths in an unvarnished and simple guise. Ever satisfied that our success should be commensurate with our merits, we do not shrink from impartial comparison with our much respected sister institutions. On the part of this institution be assured, that as our star is in the ascendant, so is it the deliberate determination of the faculty of this university to maintain it in a position second to none in the country. In many things essential to an elevated standard of medical education, the founders of this college claim to have led the way in reform and improvement. Convinced that by increasing the facilities for a more thorough and efficient course of instruction, as well as by making a high standard of attainment requisite to the reception of its honors, the true interests of the student and school are alike consulted; our course, hereafter as heretofore, shall be onward and upward.

Actuated by such motives, encouraged and sustained by such demonstrations as are now before me, can there be any doubt that the same talent and industry, by persevering in the course already so successfully pursued, will bear aloft the reputation of this institution, and enable you ever with filial pride and exultation to point to the elevated position of your *Alma Mater.*

But, to proceed in the duties of admonition and counsel. The first caution which I would give you, in the pursuit of your professional avocation is, to avoid quackery yourselves. For, I regret to be compelled to admit, that quackery is not entirely confined to the unlearned; there are those "who know the right, but still the wrong pursue." The forms which this *hydra headed* monster assumes are endless. The material out of which they are evolved is, however, essentially the same in all ages and countries. Certain medical errors, which are common to man everywhere, constitute its basis.

When some men come to the subject of medicine they exercise their credulity only, laying aside all those tests which they commonly apply to sentiments and opinions for their analysis. Even those who exhibit great stability and acuteness of mind, sifting evidence and rejecting plausible error on other subjects, are often seen to be "blown about by every wind of doctrine" in medicine. We, therefore, console ourselves with the reflection, so far as the laity is concerned, that it arises in part, at least, from a peculiar fondness, which exists in some minds, for the marvelous, and that those who run after nostrums and the various isms of the moment here, would, in other countries, impelled by the same blind faith and ignorance, make their Meccan pilgrimages to kiss and bow down to consecrated relics.

Do not, however, resort to denunciation as a remedy for this evil, when you encounter it in your professional pursuits. This would but confirm ignorance in obstinacy and lend its advocates another weapon by raising the cry of persecution. Seek rather to communicate information on the various subjects which distinguish science from quackery, enable them to look through the shallow covering which conceals its deformities and perceive that *their* interests and not *yours* are involved in the question. Remember the opinion enunciated recently on a similar occasion by my able colleague, Prof. Moore, "that men of science are rarely the victims of quackery," and instil into their minds scientific principles as opportunity is afforded.

Although occasionally an old prejudice against one mode of practice, or against one class of remedies or another, is revived and obtains an ephemeral popularity; still do we believe that the march of public confidence in scientific acquirement is steadily onward, and that it advances exactly in proportion, as the mass of community itself becomes enlightened. To this general rule, I know of but one exception, and that pertains to that most responsible of all professions, the clergy. It is doubtless true, that no class of men have done more harm by giving certificates of cures to quack medicines, than clergymen. Fearful as the responsibility which they thus assume in encouraging heterodox opinions certainly is, we can find much charity for their error. We must bear in mind that mingling little in the world they are proverbially credulous. Hence, sympathizing with the apparently relieved sufferers, whom they meet in the discharge of their parochial duties, they listen to their glowing narrations of their supposed cures; and, without examining their statements, are induced often to furnish certificates to the shameless pretender. Again, aware of the value of these certificates in giving currency to their panaceas, the impostor importunes the influential divine for his name with a pertinacity not easily dismissed, and a reluctant assent is often yielded, forgetful that the lives of their fellow beings are but too often the sacrifice. Without dwelling longer upon this point, I will leave all unacquainted with medical science, merely citing for their reflection the remark of one of the most learned and devout men who has ever lifted the voice of warning from an English pulpit. He says: *"I have often times, not without wonder and indignation, observed the strange confidence of empyrics in physic, that dare venture on the practice of that noble art which they do not at all understand, considering how, for a little paltry gain, they shrewdly hazard or rather certainly destroy the health and lives of men; and I have judged them worthy of as capital and ignominious punishment as those that kill men on the highway."

But, though we may be inclined to regard these opinions, when advanced by those out of the pale of the profession, with great leniency, and pity the evil consequences which must sooner or later follow from

*Bishop Bull.

their propagation, no palliation can be claimed for *professional* unfaithfulness. To the treacherous brother who, possessed of all the light necessary to see the deep abyss into which he unhesitatingly plunges all those who confide in his sagacity, no hope of extenuation or mercy can be extended. Can he ask for our charitable opinion who, lured on by no other motives than the desire of completing a successful career of deception by amassing a princely fortune at the expense of human life, with a full knowledge of the terrible dangers to which thousands must be subjected, to accomplish his nefarious purposes, can *he* ask us to witness, unrebuked, this effort to commend the poisoned chalice to the lips of his unsuspecting fellow mortal? But, gentlemen, I trust there can be no danger of such unmitigated defection, of the commission of such a flagrant sin under the rays of a full blaze of light.

There is, however, a more insidious form of quackery against which the younger members of our profession require to be cautioned. It pertains to our *professional* intercourse as well as to our intercourse with our patients. Where this *spirit of quackery* exists, it will be manifested in concealed efforts to promote our own advancement by unfavorable comparisons instituted concerning other practitioners, prompting to cunning arts to deceive the public, and it is in nothing more frequently observed than in an over-weening desire for reputation, with an indifference to the grounds upon which it is based, producing a competition among physicians resting, often, upon issues which are utterly false. Under such circumstances medicine is followed as a mere trade. Neither the public welfare or the development of truth is made prominent in pursuit; and credulity is fostered, furnishing ample oportunity for the dissemination of empyricism. The eradication of this tendency to a base self-aggrandisement is a work of reform which commends itself to your earnest consideration. Let competition always be honorable, without descending to the low arts of the mere pretender. Remember, with classes as with individuals, it is impossible to secure the respect of the community for the profession unless we preserve our own honor and self-respect. Lay aside all narrow and selfish motives, and cultivate that *esprit du corps* which will lead you to act in unison for the good of the profession, as a whole. By respecting ourselves and the reputation and honor of other members of the faculty, only, can we hope to inspire public confidence.

The community have not very correct ideas of the rules of honorable intercourse among physicians, and are frequently disposed to cast contempt upon all observance of medical etiquette. Be not tempted into an infraction of these rules, however, in consequence of the obtuseness of public sentiment on this subject. Rely upon it, these are alike necessary for yourselves and for securing the safety of your patient. Let these necessary rules, then, as adopted by the great American Medical Association, be engraven on your forehead. Think of them when you rise up and when you lie down, and when in the highway of your professional labors, and observe them with scrupulous exactness. Remember that you go forth to do battle against empyricism, honoring your *alma mater*, and securing to yourselves, I trust, well merited riches, and a good name, more precious than rubies. Aye, a good name! But do not flatter yourselves that your utmost efforts to deal justly, your untiring vigilance to govern yourselves in your professional intercourse, by the strictest rules of medical ethics, will effectually secure you against the envenomed shafts of jealousy and envy. No! be not disappointed! Success is certain to call down upon you unmerited and unmeasured scandal, which is the only weapon within the reach of little minds, and of which it is a certain indication. But, gentlemen, permit me to enjoin you never to allow yourselves to be goaded into retaliation. Bear in mind that the honor and dignity of the profession are at stake, and that nothing can detract so much from the confidence which the public extend to it and through it to yourselves, as professional bickerings and jealousies. Detraction will but lend increased strength to empyricism, and drag those who indulge in it to a level scarcely above the unblushing charlatan. Never yielding to despair, always place before you high scientific attainment as the only end worthy your ambition in this world; and, in an honest and persevering effort in its accomplishment, you will find little time or inclination to turn aside for the purpose of refuting any of the vile machinations which a successful course is sure to excite against you. Besides, at the very best, this were but labor lost, for truth is mighty and must sooner or later prevail, and seldom will it occur that a slanderous device does not recoil upon the head of its inventor; and the opinion of the virtuous and reflecting, in the form of retributive justice, suspends him upon that gallows which he had erected for another.

Remember, then, that the profession of your choice has claims upon you which you cannot honorably disregard, and that your true course of policy and peace of mind lie in the same direction; and, above all, remember that this is the rule given by Him who, when he was reviled, reviled not again; remember, I say, all these things, and never allow yourself to be provoked to speak harshly of your erring, misguided, thoughtless, but envious professional brother.

It can scarcely be necessary that I remind you of the importance of temperance and sobriety, of your obligations to secrecy, discretion and honor. Into your hands the husband and father entrust those whose honor is more dear to them than life, and which you must sacredly preserve. Into your keeping will be confided the occurrences of the sick room, where all restraint is thrown aside, admitting you into the inmost sanctum of their hearts; hence the first step in the study and the closing act in the practice of medicine should find your lips sealed.

Nor can it be needful that I dwell upon the importance of attending to your address and manners, so that they may be liberal and polished. That in your intercourse with the sick you be compassionate and gentle, bearing the contradictions, complaints and disappointments incident to their infirmities with equanimity. Often will you be required to gratify whims and caprices of the most unreasonable character. Comply with them with apparent cheerfulness wherever you can do so with propriety; but never allow your flexibility to induce you to consent to that which, in your opinion, is contrary to the interests of your patients, however pleasing it might be to their inclinations. Too great pliancy not only endangers the immediate welfare of the patient, but lessens confidence and is succeeded often by contempt and estrangement.

In the familiar intercourse which almost necessarily obtains between the physician and his patient, what an infinite responsibility does he incur in the principles which he inculcates in the families of his charge. How necessary that he should be careful in the expression of his opinions. At what high ends should he aim by his daily example. How important that he should be right in the great moral questions of the day which agitate the community, and that his morality should be strictly that of the Bible. And when he enters the sacred precincts of the sick chamber, how important that sincerity and candor should characterize his every step. The issues of life and death are before him; he, and perhaps he only, has communication with the spirit in its most momentous hours, and under such circumstances, with no clerical friend, no pious relative near to advise, there is truly a fearful responsibility resting upon him. Should such opportunities be employed to the well-being of those falling under his influence, what an infinite source of happiness is offered to the truly benevolent physician in the opportunity thus presented for exerting a good moral and religious influence. How infinitely superior to any pecuniary compensation would be the delightful reflection that through his instrumentality the abode of misery and vice has been converted into one of virtue and peace. Especially when he is permitted to enjoy as the reward of his efforts the happy thought that through his influence and counsel he has been instrumental in saving the soul as well as the body from death, has he a higher joy than success, however brilliant, and honor, however profusely awarded, and gratitude, however ardent, can impart to his soul.

In conclusion, then, permit me to add that, scattered as you soon will be, whither your duty or inclination calls you, to the north or to the south, to the east and the west, over this widespread country, let me affectionately solicit, for my colleagues and for myself, that your thoughts may occasionally revert to those whose delightful office it has been to guide you in your preparatory labors, and whose pride and pleasure it has been to remove from your path all obstacles to your onward progress. In the name of the council and faculty of the University of Buffalo, I welcome you to the ranks of the profession of which you have this day received your testimonials as accepted members. In their name, and in the name of that profession, I exhort you to follow the dignified calling you have embraced, with zeal, circumspection, and honor. Recommending you to shun idleness and make work attractive; adopting the motto that all who would win must labor for the prize; and in the belief that there is much truth in the maxim that God will help those who help themselves; and that no standard of attainment or success is too elevated when sought by patient and enduring toil; and commending you to the divine blessing for happiness here and hereafter, I bid you all an affectionate farewell.

